# Enhancing Psychiatric Crisis Response for Foreign Travelers: Addressing Suicide Risk through Cross-border Continuity of Care

**DOI:** 10.31662/jmaj.2025-0212

**Published:** 2025-08-29

**Authors:** Taro Wakutsu, Soichiro Saeki

**Affiliations:** 1 National Center for Global Health and Medicine, Japan Institute for Health Security, Tokyo, Japan; 2 Division of Public Health, Department of Social Medicine, Graduate School of Medicine, The University of Osaka, Osaka, Japan

**Keywords:** suicide attempts among tourists, emergency protocols, preventative interventions, foreign travelers

Yamashita et al. ^(1)^ showed that loneliness, intensified by cultural isolation and systemic barriers during the coronavirus disease 2019 pandemic, is independently associated with depressive and anxiety symptoms among Vietnamese migrants in Japan. A comparable concern arises in the context of foreign travelers with pre-existing psychiatric disorders, an underexplored demographic in the current literature ^(2)^. Suicidal behavior is not confined to long-term migrants or international students; it can also manifest among transient populations exposed to sudden cultural, environmental, and psychological disruptions.

Previous studies have primarily focused on the phenomenon of “suicide tourism,” whereby individuals travel with explicit suicidal intent. However, little is known about the clinical characteristics and contextual factors surrounding suicide attempts by travelers without prior suicidal ideation ^(2)^. Such individuals―whether well-managed or struggling with underlying psychiatric conditions―may travel seeking emotional respite, novelty, or distraction. Nonetheless, the unpredictability of travel schedules, crowded destinations, overstimulating environments, and the intensity of touristic activities may exacerbate psychological distress ^(3)^. Furthermore, being abroad may severely limit access to regular psychiatric care and essential medications, whereas stigma and language barriers may delay timely intervention.

Continuity of psychological care across borders represents an additional challenge. Within Japan, reliance on physical referral letters complicates care transitions, an issue magnified in an international context. This dependence on patient-mediated information transfer imposes an undue burden on individuals, risking treatment discontinuity.

The literature gap highlights the urgent necessity of establishing a globally responsive mental healthcare framework that ensures equitable psychiatric emergency care, regardless of nationality. For foreign travelers, the absence of accessible medical histories and medications delays interventions and increases suicide risk. Language barriers are also a pervasive obstacle ^(4)^, linked to delayed care-seeking and worsened clinical outcomes.

Efficient cross-border sharing of critical psychiatric information, such as history and medication regimens, should be integral to emergency response systems. For example, initiatives such as MyHealth@EU (https://health.ec.europa.eu/ehealth-digital-health-and-care/digital-health-and-care/electronic-cross-border-health-services_en) in the European Union and the HL7 International Patient Summary (https://build.fhir.org/ig/HL7/fhir-ips/) provide models for international information sharing. Adoption of such a framework can meaningfully reduce treatment disruptions and enhance continuity of psychiatric care during acute crises and on repatriation.

In conclusion, reducing the risk of suicide among international visitors with mental health vulnerabilities and creating an inclusive tourism management model requires standardized international protocols for psychiatric emergencies, encouraging intersectoral collaboration ^(5)^, and enhancing international medical information sharing. In addition to protecting those who are vulnerable, establishing such inclusive treatment models will mark a critical advancement in global mental health governance.

## Article Information

### Conflicts of Interest

None

### Acknowledgement

The authors thank their colleagues for helpful discussions on this topic. The authors acknowledge the use of Grammarly (Grammarly Inc, San Fransico, USA) for primary language editing. The views expressed in this manuscript are those of the authors and do not necessarily represent the authors’ institutions.

### Author Contributions

Taro Wakutsu prepared the first draft of the manuscript. Soichiro Saeki critically reviewed the manuscript. Both authors read and approved the final version of the manuscript. Artificial intelligence technology was used for the language editing process, and the authors reviewed such content. The authors’ institutions played no role in the conceptualization of this manuscript.

### Approval by Institutional Review Board (IRB)

Not applicable.
